# A19 THE IMPACT OF FOOD INSECURITY ON ADHERENCE TO A GLUTEN-FREE DIET IN THE ADULT CELIAC DISEASE POPULATION ATTENDING A DEDICATED CELIAC CLINIC

**DOI:** 10.1093/jcag/gwad061.019

**Published:** 2024-02-14

**Authors:** R Leong, S Tandon, M Khaouli, J Blom, R Daca, E Verdu, D Armstrong, M Pinto-Sanchez

**Affiliations:** McMaster University, Hamilton, ON, Canada; Medical Sciences, McMaster University Faculty of Health Sciences, Hamilton, ON, Canada; McMaster University, Hamilton, ON, Canada; McMaster University, Hamilton, ON, Canada; McMaster University, Hamilton, ON, Canada; Medical Sciences, McMaster University Faculty of Health Sciences, Hamilton, ON, Canada; Medicine, McMaster University Faculty of Health Sciences, Hamilton, ON, Canada; Medical Sciences, McMaster University Faculty of Health Sciences, Hamilton, ON, Canada

## Abstract

**Background:**

A strict gluten-free diet (GFD) is currently the only treatment for celiac disease (CeD), but a GFD can be inconvenient and expensive. CeD patients who are food insecure (FI) face threats to GFD adherence, overall health, and quality of life.

**Aims:**

To evaluate the prevalence of FI in patients with CeD attending a dedicated Adult Celiac Disease Clinic (ACDC) at an academic hospital and to examine the relationship between FI and GFD adherence, quality of life and various gastrointestinal symptoms.

**Methods:**

Patients were invited to participate in the CeD registry when they attended the ACDC; those who agreed to participate then provided verbal and signed consent. Patients had the option to complete the CeD registry forms in the clinic on an iPad or at home using a card with a QR code and a unique ID to access the online CeD registry. The CeD registry uses REDCap (v. 11.1, 2021, US) and includes six validated questionnaires (Celiac Diet Adherence Test (CDAT), Celiac Disease Quality of Life Questionnaire (CD QoL), Celiac Symptom Index (CSI), Gastrointestinal Symptom Rating Scale (GSRS), Hospital Anxiety and Depression Scale (HADS), and Household Food Security Survey Module (HFSSM)). Continuous data are expressed as mean (SD), and categorical data as proportions of patients. The chi-squared test with Fisher correction and *t*-test were used to assess differences between groups.

**Results:**

A total of 653 patients attended the ACDC from November 2, 2022, to September 20, 2023, and 411 patients were invited to participate in this study. Of them, 204 completed the CeD registry. Food insecurity was identified in 16% of CeD patients, of whom 11 (5%) were marginally FI, 16 (8%) were moderately FI, and 7 (3%) were severely FI. Compared to CeD patients who are food secure, a greater proportion of FI CeD patients reported nonadherence to GFD (26/34 vs 70/170; p<0.001), reduced QoL (32/34 vs 98/170; p<0.001), greater anxiety (27/34 vs 84/170; p=0.001) and greater depression (13/34 vs 26/170; p=0.004) compared with normal values. CeD patients with FI also reported lower QoL [61.0(14.9) vs 49.6(16.7); p=0.001] and increased gastrointestinal symptoms [GSRS 37.7(14.7) vs 29.0(12.8); p=0.001] compared with food secure CeD patients.

**Conclusions:**

Food insecurity is common in patients with CeD and is associated with significantly worse adherence to treatment, symptom control, and quality of life. There is an important need for programs and societal measures to abolish food insecurity and ensure adequate access to treatment for patients with CeD.

**Funding Agencies:**

TRIANGLE Summer Studentship

